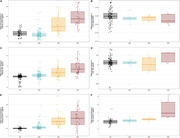# Longitudinal plasma and CSF analyses in Down syndrome. Results from the DABNI cohort

**DOI:** 10.1002/alz.092030

**Published:** 2025-01-09

**Authors:** Daniel Alcolea, Laia Montoliu‐Gaya, Maria Carmona‐Iragui, Isabel Barroeta, Laura Videla, Bessy Benejam, Javier Arranz, Alberto Lleo, Kaj Blennow, Nicholas J. Ashton, Henrik Zetterberg, Juan Fortea

**Affiliations:** ^1^ Sant Pau Memory Unit, Hospital de la Santa Creu i Sant Pau, Biomedical Research Institute Sant Pau, Universitat Autònoma de Barcelona, Barcelona Spain; ^2^ Center of Biomedical Investigation Network for Neurodegenerative Diseases (CIBERNED), Madrid Spain; ^3^ Department of Psychiatry and Neurochemistry, Institute of Neuroscience and Physiology, The Sahlgrenska Academy, University of Gothenburg, Mölndal Sweden; ^4^ Barcelona Down Medical Center, Fundació Catalana Síndrome de Down, Barcelona Spain; ^5^ Hospital de la Santa Creu i Sant Pau ‐ Biomedical Research Institute Sant Pau ‐ Autonomous University of Barcelona, Barcelona, Catalonia Spain; ^6^ Centre of Biomedical Investigation Network for Neurodegenerative Diseases (CIBERNED), Madrid Spain; ^7^ Clinical Neurochemistry Laboratory, Sahlgrenska University Hospital, Mölndal Sweden; ^8^ NIHR Biomedical Research Centre for Mental Health & Biomedical Research Unit for Dementia at South London & Maudsley NHS Foundation, London UK; ^9^ Wallenberg Centre for Molecular and Translational Medicine, University of Gothenburg, Gothenburg Sweden; ^10^ Department of Old Age Psychiatry, Institute of Psychiatry, Psychology, and Neuroscience, King’s College London, London, London UK; ^11^ Department of Psychiatry and Neurochemistry, Institute of Neuroscience and Physiology, The Sahlgrenska Academy, University of Gothenburg, Mölndal, Gothenburg Sweden; ^12^ Hong Kong Center for Neurodegenerative Diseases, Hong Kong China; ^13^ Department of Neurodegenerative Disease, UCL Queen Square Institute of Neurology, University College London, London, ‐ UK; ^14^ UK Dementia Research Institute at UCL, London UK

## Abstract

**Background:**

Alzheimer´s disease (AD) is the main cause of death in adults with Down syndrome (DS). We describe the unique contributions of the Down Alzheimer Barcelona Neuroimaging Initiative (DABNI) cohort by studying longitudinal changes in plasma and cerebrospinal fluid (CSF) markers.

**Method:**

We included DABNI participants with DS that contributed at least two plasma and/or CSF samples and were asymptomatic (aDS, n=155), had prodromal AD (pDS, n=46) or had AD dementia (dDS; n=53) at baseline, together with 172 euploid cognitively normal controls (CN). All participants underwent longitudinal assessments, including repeated cognitive and neuropsychological assessments, plasma and CSF analyses. We applied linear‐mixed models to study changes over time in measures of GFAP, NfL and pTau217 in plasma and in AB42/AB40, NfL and pTau217 in CSF and their association with the symptomatic stages of AD.

**Result:**

Plasma GFAP and NfL showed higher increases in dDS compared to pDS, and in pDS and dDS compared to aDS and CN (Figure 1A and 1B). Plasma pTau217 showed higher increases in pDS and dDS compared to aDS and CN, but there were no differences between pDS and dDS (Figure 1C). In CSF, no significant longitudinal changes were detected in AB42/AB40 (Figure 1D), whereas both NfL and pTau217 showed higher slopes in advanced symptomatic stages (Figure 1E and 1F).

**Conclusion:**

Our results indicate that longitudinal changes in plasma markers are associated to different stages in the continuum of AD in DS. These changes could be useful to track the progression of the disease.